# Biomarkers for Duchenne muscular dystrophy progression: impact of age in the *mdx* tongue spared muscle

**DOI:** 10.1186/s13395-023-00325-z

**Published:** 2023-09-13

**Authors:** Marcelo dos Santos Voltani Lorena, Estela Kato dos Santos, Renato Ferretti, G. A. Nagana Gowda, Guy L. Odom, Jeffrey S. Chamberlain, Cintia Yuri Matsumura

**Affiliations:** 1 Department of Structural and Functional Biology, Institute of Biosciences, São Paulo State University (UNESP), Botucatu, São Paulo, Brazil; 2 Anesthesiology and Pain Medicine, Northwest Metabolomics Research Center, Mitochondria and Metabolism Center, University of Washington, Seattle, WA USA; 3 Department of Neurology, Wellstone Muscular Dystrophy Specialized Research Center, University of Washington School of Medicine, Seattle, WA USA

## Abstract

**Background:**

Duchenne muscular dystrophy (DMD) is a severe form of muscular dystrophy without an effective treatment, caused by mutations in the *DMD* gene, leading to the absence of dystrophin. DMD results in muscle weakness, loss of ambulation, and death at an early age. Metabolomics studies in *mdx* mice, the most used model for DMD, reveal changes in metabolites associated with muscle degeneration and aging. In DMD, the tongue muscles exhibit unique behavior, initially showing partial protection against inflammation but later experiencing fibrosis and loss of muscle fibers. Certain metabolites and proteins, like TNF-α and TGF-β, are potential biomarkers for dystrophic muscle characterization.

**Methods:**

To investigate disease progression and aging, we utilized young (1 month old) and old (21–25 months old) *mdx* and wild-type tongue muscles. Metabolite changes were analyzed using 1H nuclear magnetic resonance, while TNF-α and TGF-β were assessed using Western blotting to examine inflammation and fibrosis. Morphometric analysis was conducted to assess the extent of myofiber damage between groups.

**Results:**

The histological analysis of the mid-belly tongue showed no differences between groups. No differences were found between the concentrations of metabolites from wild-type or *mdx* whole tongues of the same age. The metabolites alanine, methionine, and 3-methylhistidine were higher, and taurine and glycerol were lower in young tongues in both wild type and *mdx* (*p* < 0.001). The metabolites glycine (*p* < 0.001) and glutamic acid (*p* = 0.0018) were different only in the *mdx* groups, being higher in young *mdx* mice. Acetic acid, phosphocreatine, isoleucine, succinic acid, creatine, and the proteins TNF-α and TGF-β had no difference in the analysis between groups (*p* > 0.05).

**Conclusions:**

Surprisingly, histological, metabolite, and protein analysis reveal that the tongue of old *mdx* remains partially spared from the severe myonecrosis observed in other muscles. The metabolites alanine, methionine, 3-methylhistidine, taurine, and glycerol may be effective for specific assessments, although their use for disease progression monitoring should be cautious due to age-related changes in the tongue muscle. Acetic acid, phosphocreatine, isoleucine, succinate, creatine, TNF-α, and TGF-β do not vary with aging and remain constant in spared muscles, suggesting their potential as specific biomarkers for DMD progression independent of aging.

**Supplementary Information:**

The online version contains supplementary material available at 10.1186/s13395-023-00325-z.

## Introduction

Duchenne muscular dystrophy (DMD) affects approximately 1 in 3600–6000 male live births [1] and is a severe form of muscular dystrophy without an effective treatment [2]. It is an X-linked recessive disease [2] caused by mutations in the *DMD* gene [3] which leads to the absence of dystrophin [4].

The lack of dystrophin causes continuous loss of muscle strength, myofiber damage, chronic inflammation, progressive fibrosis, and muscle stem cell dysfunction. This dystrophic scenario leads to a loss of ambulation in the early teens to 20s. Although patients’ life expectancy has improved with current standards of cardioprotective care and respiratory support, they often die around the third or fourth decade of life, mainly due to cardiac and respiratory complications [2, 5, 6].

The *mdx* mice is the most used animal model for research on DMD [7, 8]. Around 20 days old, these animals begin to show their first signs of muscle degeneration and regeneration [9]. The *mdx* mice have reduced life spans, by about 17 to 19% compared to wild type. At 26 months, average lifespans, the muscles present typical dystrophic characteristics: loss of muscle fibers with increased fibrosis, fat infiltration, necrotic fibers, and regenerated fibers [10].

Metabolomics are the quantitative analysis of metabolites produced by an organism under certain conditions. Metabolomics provide an integrated view of biochemical pathways in complex organisms, thus producing a more detailed and systematic overview of the cellular processes and its response to diseases. Therefore, this approach is essential for the definition of personalized medicine, through the establishment of metabolite profiles and biomarkers for certain pathological states [11]. Studies in *mdx* mice show changes in metabolites related to the progression of muscle degeneration and aging [12–14].

In DMD, the muscles of the oral cavity are also affected, causing dysphagia in late stages of the disease, which worsens with advancing age and disease progression [15, 16]. In *mdx* mice, the tongue muscles show an unusual behavior as the disease progresses compared to other muscles. In 3-month *mdx* mice, inflammatory cells were hardly found in the tongue, unlike other masticatory muscles. In addition, the expression of collagen did not change in the tongue of these *mdx*, while it was three times higher in masseter muscle, when compared to the control [17]. These results indicate partial protection of this muscle against myonecrosis and inflammation at 3 months of age. However, at 23 months of age, foci of inflammation and fibrosis were identified [18]. Additionally, Chamberlain et al. (2007) [10] described the tongue of the *mdx* as the second most affected muscle at 26 months of age, due to fibrosis and the loss of fibers in the central portion of the muscle, just after the diaphragm.

Some metabolites from dystrophic mice were analyzed by nuclear magnetic resonance (NMR) in different types of tissues and have been suggested to determine biomarkers for the state of muscle fibers [19]. Martins-Bach and colleagues [13] studied the diaphragm and quadriceps muscles, in 3-month and 6-month-old dystrophic and non-dystrophic mice, and found that the metabolites glutamate, glutamine, succinate, isoleucine, acetate, alanine, glycerol, carnosine, taurine, glycine, methionine, and creatine had alterations when compared to same age controls and also across time [13]. Griffin and colleagues [20] studied cardiac, cortex, cerebellum, diaphragm, and soleus tissue and stated that different ratios of the founding metabolites were responsible to distinguish the dystrophic and non-dystrophic tissue, including the following: phosphocholine/taurine, glutamate/creatine, and taurine/creatine [20]. Taurine itself has already been indicated as a biomarker for dystrophin deficiency in the quadriceps muscle, liver, and kidney [21].

Besides metabolites, there are some proteins that are well established in the current literature related to inflammation and fibrosis, like tumor necrosis factor *alpha* (TNF-α) and transforming growth factor *beta* (TGF-β), respectively. These proteins are very promising biomarkers for the dystrophic muscle characterization, for their relation to the myonecrosis as seen in the *mdx* animal model and human patients [22]. Given tongue’s unusual behavior within DMD progression, being spared in young mice and severely affected in old mice [10, 17], we evaluated metabolites and proteins in the tongue muscles, within young (30 days old) and old (21 to 25 months old) *mdx* and wild-type mice. Since the proteins and metabolites chosen were previously reported as possible biomarkers for the characterization of the dystrophy pathology and progression, in muscle and other tissues [13, 19–27], the present study aimed to validate these biomarkers in the tongue of *mdx* mice over time. Surprisingly, our histological results found that the tongue remains partially spared in older *mdx* mice (21 to 25 months). Our results allowed us to identify biomarkers that change with aging, regardless of the absence of dystrophin. Other biomarkers seem to be excellent candidates to indicate the progression of dystrophinopathy over time, as they do not change with aging and remain constant in the spared muscles. Furthermore, analyzing these possible biomarkers in the tongue at different ages can help to understand the protection mechanisms involved in the pathological process and support the development of future approaches for the diagnosis and monitoring progression of DMD.

## Materials and methods

### Animals

Male and female, young and old *mdx* (C57BL/10-DMD^*mdx*^/PasUnib), and age-match wild-type mice (C57BL/10ScCr/PasUnib) were obtained and maintained by our institutional animal care facility of Institute of Bioscience (Botucatu) — UNESP. All mouse experimentation was approved by our institution committee and done in accordance with the guidelines of the Brazilian College for Animal Experimentation (protocol n° 1095-CEUA). The animals were divided into four groups: young *mdx* (1 month old), old *mdx* (21–25 months old), young wild type (1 month old), and old wild type (21–25 months old).

### Tissue harvesting

The animals were euthanized with an overdose of intraperitoneal anesthesia of xylazine hydrochloride (30 mg/kg) and ketamine hydrochloride (300 mg/kg). The tongue was dissected and fixed for histological techniques or frozen in liquid nitrogen for Western blotting and metabolome assays.

### Histology

The mid-belly tongue muscles of the young wild type (*n* = 5), young *mdx* (*n* = 5), old wild type (*n* = 4), and old *mdx* (*n* = 6) groups were sectioned and stained with Masson’s trichrome, to distinguish and quantify the areas of fibrosis (FIB) and areas of muscle fibers with peripheral nuclei (PN) and central nuclei (CN). The analyses were performed blindly, and the areas were expressed in relation to the area of total transverse fibers of the section.

### Proteins analysis

Proteins related to the mechanisms of inflammation (TNF-α) and fibrosis (TGF-β) were quantified in the whole tongue with the Western blotting assay, as described previously [28]. The values were normalized with the glyceraldehyde 3-phosphate dehydrogenase protein (GAPDH), incubated on the same membrane after routine stripping methods.

### Metabolomics by nuclear magnetic resonance (NMR) spectroscopy

In order to verify possible changes in the metabolic profile, metabolomics analysis was performed by NMR spectroscopy of the whole tongue muscles of young (*n* = 10) and old (*n* = 5) wild-type mice and young (*n* = 11) and old (*n* = 5) *mdx* mice.

Data acquisition was performed on a Varian INOVA spectrometer operating at a resonance frequency of 1 H of 600 MHz. The samples were homogenized in a methanol/chloroform solution (2:1). After 15 min, a solution of chloroform/Milli-Q water (1:1) was added to the pellet. Then, the samples were centrifuged at 4000 RPM for 20 min. The supernatant was collected and lyophilized. The obtained powder was resuspended in deuterated water (D_2_O) with trimethylsilyl tetradeuteropropionic acid (TSP, 10 mM). Subsequently, the samples were transferred to a standard NMR tube for spectral analysis. The D_2_O allowed the device to be monitored and blocked by the device’s resonant frequency. The TSP reference signals were used to assess the quality of the spectra as well as to quantify the identified substances. Proton spectra in one dimension, using pulse sequences, optimized for suppression of the water signal and were collected at 25 °C [29].

Spectrum treatment and identification and quantification of metabolites were performed using the Bruker Topspin 3.1 and Chenomx NMR Suite (Version 7.1; Chenomx Inc., Edmonton, Canada) application packages, in conjunction with the Human Metabolome Database [30] and literature already published. All the chemical shift intervals (ppm) are listed in Table 1. In parallel with the chemometric analysis using the separation of the spectrum in small intervals, the statistical analysis was performed directly with the concentrations of the identified metabolites, through the targeted profiling methodology, developed by the creators of the Chenomx NMR Suite application. In this methodology, the groups of peaks corresponding to each metabolite are identified and quantified using a database of pure substance spectra. We also used the spectral binning methodology, in which the spectra are divided into predefined frequency intervals, while the integrals of the signals within each interval are used in the statistical analysis [31].

**Table 1:** Assignments of resonance peaks from 1H nuclear magnetic resonance (NMR) data. Areas were calculated considering the indicated chemical shift intervals

Chemical shifts interval (ppm)	Compound
1.0056–1.0283	Isoleucine
1.4639–1.5053	Alanine
1.9155–1.9262	Acetic acid
2.4002–2.4134	Succinic acid
3.0253–3.0459	Creatine
3.0458–3.0533	Phosphocreatine
3.3964–3.4469	Taurine
3.5587–3.5648	Glycine
2.6372–2.6426	Methionine
2.3322–2.3754	Glutamic acid
7.0091–7.0516	3-Methylhistidine
3.639–3.6507	Glycerol
− 0.1362–0.1198	TSP

### Statistical analysis

Statistical analysis was performed through two-way analysis of variance (two-way ANOVA, *p* ≤ 0.05) with Bonferroni’s post hoc test for histological and Western blotting data.

For the metabolomics analysis, unsupervised interactive principal component analysis (iPCA) was used in order to visualize the complex sample space and multivariate after identification and quantification of metabolites. These discriminant analyses were performed through standard procedures implemented in Pirouette 4.0 (Infometrix, WA, USA) [32]. The input variables will consist of the integral of the area intervals (spectral binning) and/or the concentration values obtained with targeted profiling. Two-way analysis of variance (ANOVA, *p* ≤ 0.05) with Bonferroni’s post hoc test was performed directly with the concentrations of the identified metabolites, using the MetaboAnalyst platform.

## Results

### Mid-belly tongue muscles remain spared in old *mdx*

**Fig. 1:**
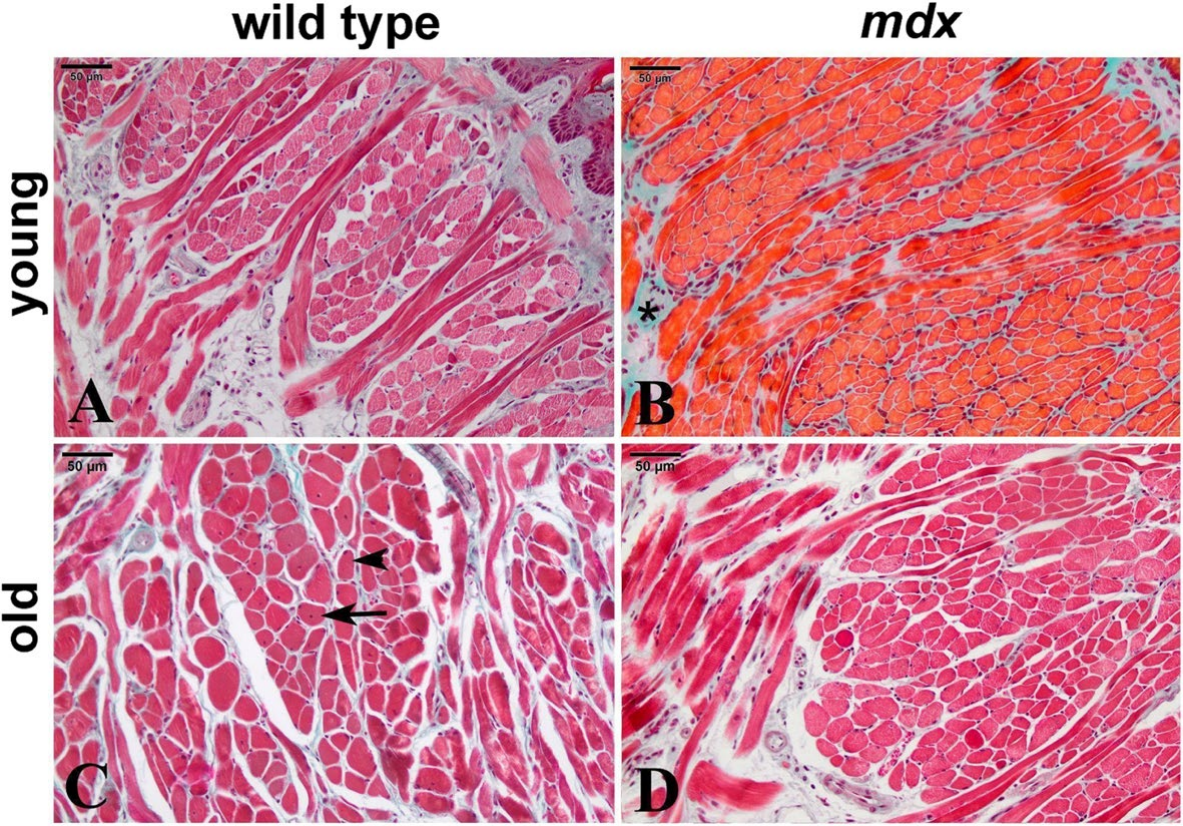
Cross sections of mid-belly tongue muscle. Young wild type (**A**), young *mdx* (**B**), old wild type (**C**), and old *mdx* (**D**) groups. In **B**, areas of fibrosis highlighted in blue by Masson’s trichrome (*). In **C**, muscle fibers with a central nucleus (arrow) and muscle fibers with a peripheral nucleus (arrowhead). Scale: 50 $\mu$m

In the qualitative histopathological analysis of the sections, we observed different histological aspects in the muscle fibers. Fibers with peripheral nuclei (PN) were observed, indicating normal muscle tissue status; fibers with central nuclei (CN), indicating regenerated muscle fibers; and areas of fibrosis (FIB), shown in blue by Masson’s trichrome stain (Fig. 1). In the quantitative histological analysis of the tongue of the groups described (Table 2), there was no difference (*p* > 0.05) in the areas of PN, CN, or FIB, both between ages, young and old, and between lineages, wild type and *mdx*.

**Table 2:** Quantitative histological analysis of the mid-belly tongue

	**%PN**	**%CN**	**%FIB**
Young wild type	91.33 ± 0.98	1.24 ± 0.63	7.42 ± 1.37
Young* mdx*	80.94 ± 13.62	2.40 ± 1.17	16.67 ± 13.30
Old wild type	83.43 ± 10.68	4.99 ± 2.77	11.58 ± 9.67
Old* mdx*	75.67 ± 5.49	6.18 ± 3.87	18.16 ± 7.29

Mean ± SD of fiber percentage areas with peripheral nucleus (%PN), central nucleus (%CN), and areas of fibrosis (%FIB) in the tongue of the young and old *mdx* and wild-type groups. There were no differences found for areas of %PN, %CN, and %FIB between the four groups (two-way ANOVA with Bonferroni’s post hoc test, *p* > 0.05)

### Changes in proteins related to inflammation and fibrosis

**Fig. 2:**
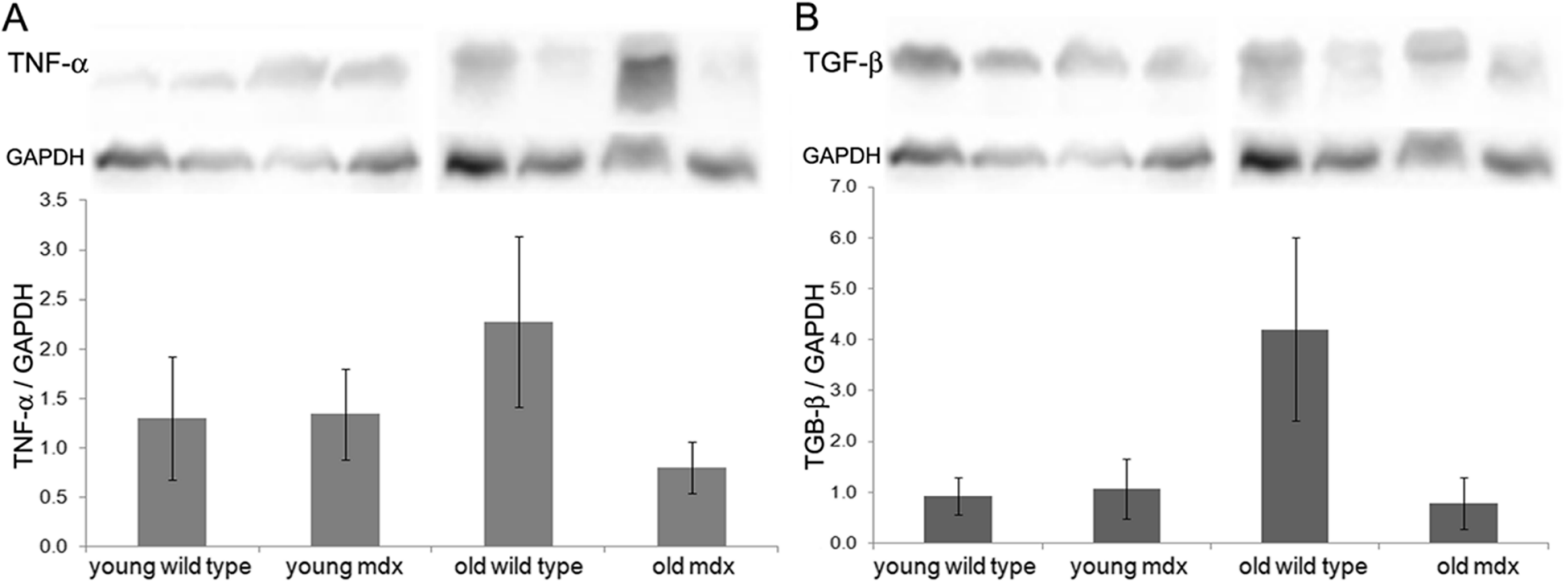
**A** Quantification of TNF-⍺ and **B** TGF-β in the whole tongue muscles. The quantification of TNF-⍺ and TGF-β was performed by Western blotting analysis in crude extracts of tongue muscles from young wild type, young *mdx*, old wild type, and old *mdx* groups. Same blot reprobed for GAPDH as a loading control. Graphs represent the level of proteins normalized to GAPDH levels expressed in arbitrary units. Bars represent standard deviation. No significant difference was observed between the groups (two-way ANOVA with Bonferroni’s post hoc test, *p* > 0.05)

The quantification of TNF-α and TGF-β in the whole tongue muscle was performed by Western blotting to verify the presence of inflammation and fibrosis, respectively. There was no significant difference in the concentrations of both proteins in the tongue between the groups (two-way ANOVA, TGF-β *p* = 9.968; TNF-α *p* = 7.558), as shown in Fig. 2.

### Changes in the metabolomic profile

**Fig. 3:**
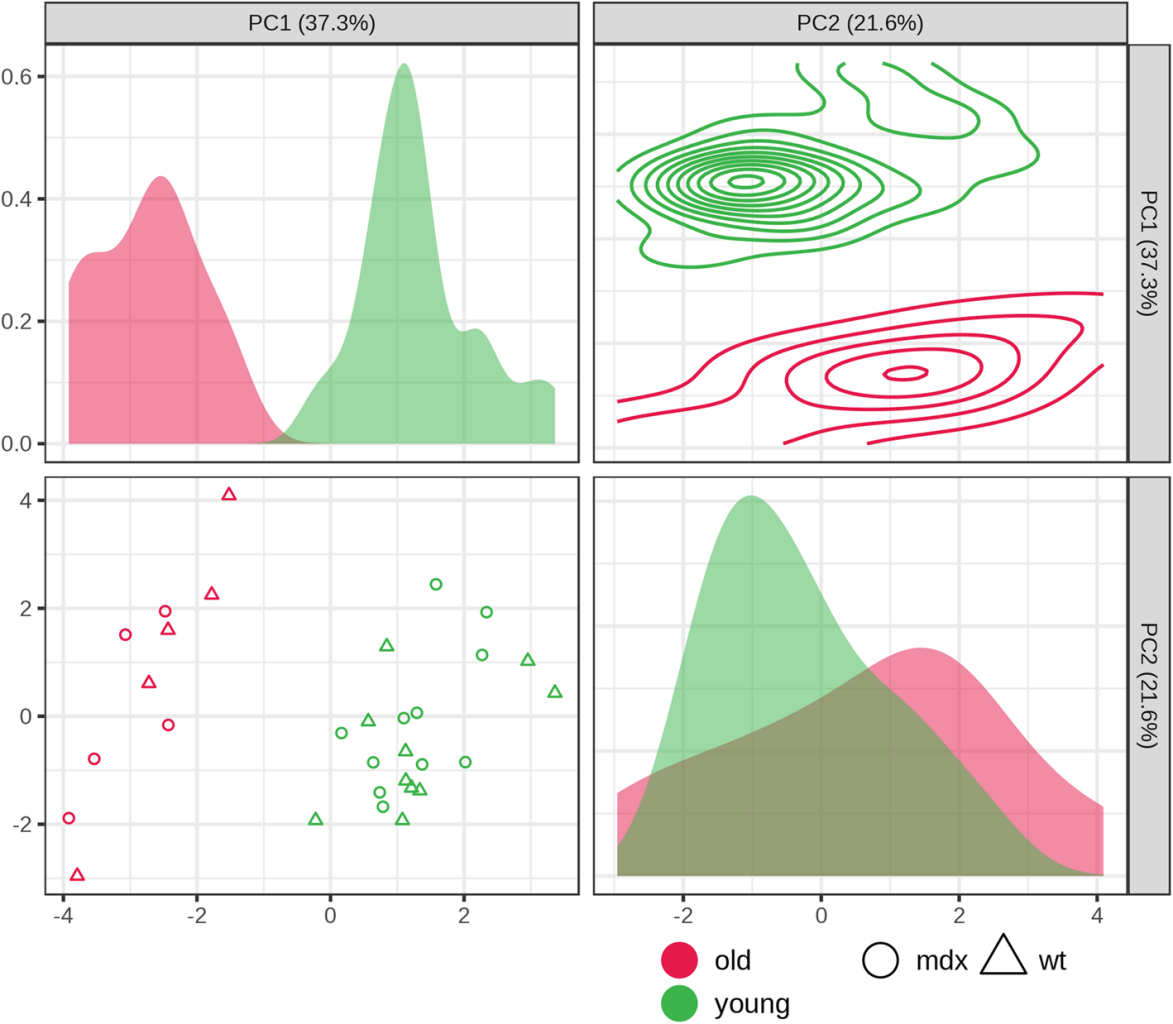
Interactive principal component analysis (iPCA) of whole tongue with the scores plot between groups. Samples spectrum plotted according to age (young, green; old, red) and genotype (mdx ◯; wild type ∆)

The whole tongues of the mice are grouped according to their metabolic profile through the iPCA. It was possible to distinguish the tongues of the same lineage at different ages; however, it was not possible to distinguish *mdx* mice from wild-type mice, in both young or old for the tongue muscle (Fig 3).

**Fig. 4:**
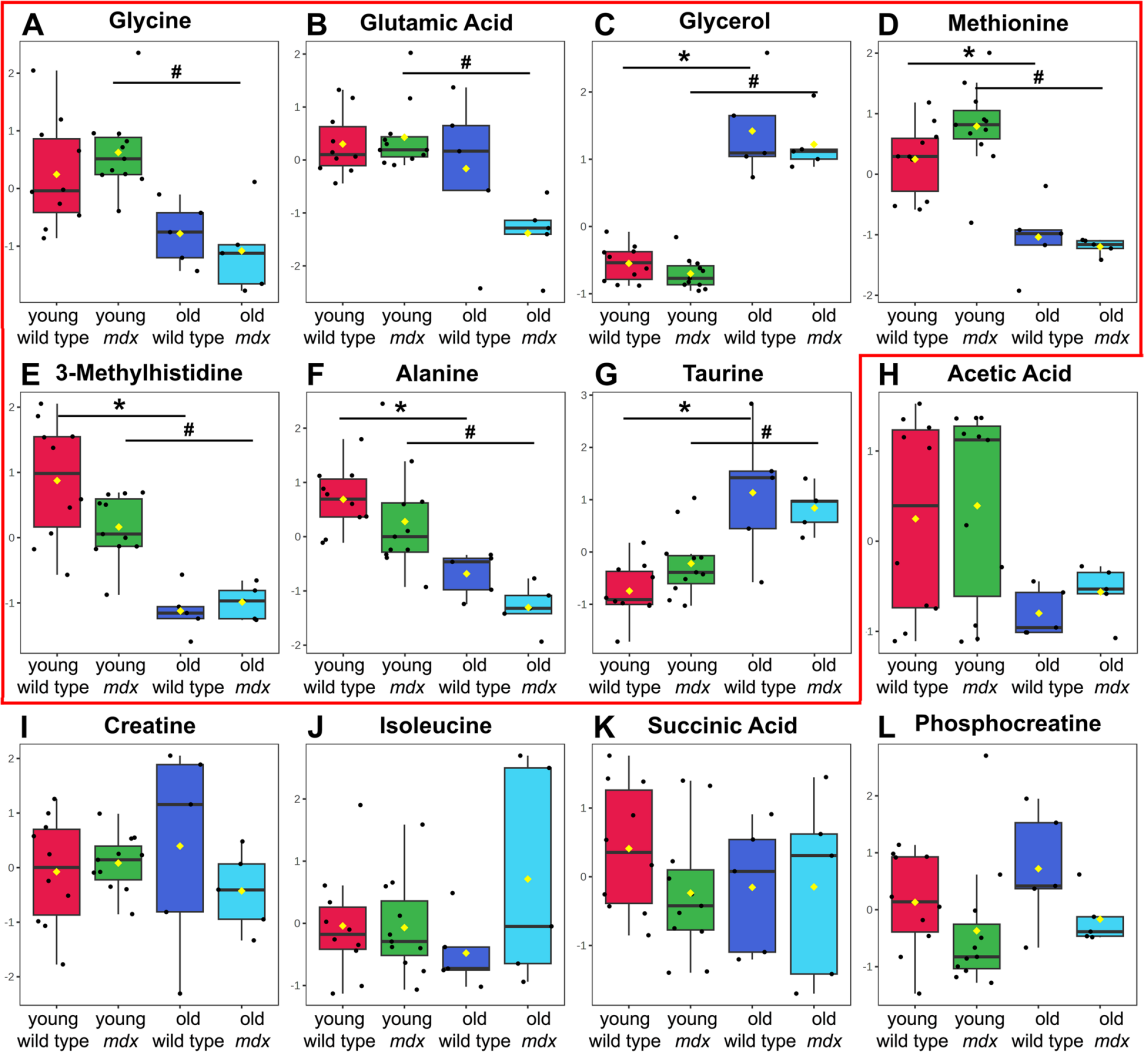
Differences in the concentrations of tongue metabolites between groups. **C**–**G** Difference between metabolite concentrations between young and old wild-type animals (*). **A**–**G** Difference between metabolite concentrations between young and old *mdx* animals (#), two-way ANOVA with Bonferroni’s post hoc test (*p* < 0.001). **H**–**L** The metabolites acetic acid, creatine, isoleucine, succinic acid, and phosphocreatine showed no differences between groups (*p* > 0.05)

No differences were found in the comparison between the concentrations of metabolites from wild-type or *mdx* whole tongue muscles of the same age, whether young or old (Fig. 4), suggesting that the protection of the dystrophic tongue muscle is observed in both ages. Table 3 summarizes the metabolites responsible for the differentiation of the tongue muscle of same strain mice at different ages. The metabolites methionine, 3-methylhistidine, and alanine were higher in young animals in both wild type and *mdx* (*p* < 0.001, Fig. 4D–F), and the metabolites taurine and glycerol were lower in both the young wild-type and *mdx* groups (*p* < 0.001, Fig. 4C and G). The metabolites glycine (*p* < 0.001, Fig. 4A) and glutamic acid (*p* = 0.0018, Fig. 4B) were different only in the *mdx* groups, being more concentrated in young *mdx* mice. The metabolites acetic acid, creatine, isoleucine, succinic acid, and phosphocreatine had no difference in the analysis between groups (*p* > 0.05, Fig. 4H–L).

**Table 3:** Different metabolites between animals of the same lineage at different ages in the whole tongue samples

	**Higher in young**	**Lower in young**
**Wild type (young vs. old)**	Alanine, methionine, 3-methylhistidine	Taurine, glycerol
***mdx*** **(young vs. old)**	Alanine, methionine, 3-methylhistidine, glutamic acid, glycine	Taurine, glycerol

## Discussion

This work aimed to validate biomarkers for diagnosis and progression of DMD through the analysis of metabolites, protein, and histology of the tongue muscles of dystrophic *mdx* mice and non-dystrophic control mice, aged 30 days (young) and 21–25 months (old).

### Dystropathology

Surprisingly, our histological results demonstrated that the mid-belly tongue of old *mdx* remains partially spared from fibrosis and myonecrosis. Previous studies from 23- and 26-month-old *mdx* mice described the tongue muscles as displaying significant histological abnormalities, including foci of inflammation and fibrosis, and localized loss of muscle fibers [10, 18]. However, in these studies, the analysis of the tongue muscle was approached qualitatively, yet without specific delineation of the particular region of the tongue analyzed. The region analyzed may interfere with the results, given the complex composition of the tongue muscle, the presence of intrinsic and extrinsic muscles by region, coupled with age-related shifts in MyHC isoform composition and differences in fiber size from the anterior, middle, and posterior regions of the tongue [33–35]. In contrast, here, the qualitative and quantitative analysis of mid-belly tongue did not show such severity. This protection was evidenced by metabolomics and Western blotting experiments performed with whole muscle of the tongue, analyzing the biomarker proteins for inflammation and fibrosis, and the concentrations of the metabolites 3-methylhistidine, acetic acid, glutamic acid, alanine, creatine, phosphocreatine, glycerol, glycine, isoleucine, methionine, succinic acid, and taurine already used for dystrophic muscle differentiation and that in this work presented similar results between wild type and *mdx*. This similarity between the chosen biomarkers was observed in young and old animals, corroborating the histological result.

Kunert-Keil and colleagues [36] report that dystrophic masseter and temporalis muscles are histologically similar to other skeletal muscles involved in the degeneration process, whereas the tongue remains with a milder phenotype. However, from the findings stated in the article, masseter and temporalis are more resistant to the calcium (Ca^2+^) overload when compared to tongue muscles of 100-day-old *mdx*. Hence, histological findings assert that inflammatory foci are hardly detectable, and dystrophic tongue contains only 11.2% of regenerated muscle, when compared to masseter, temporalis, and even soleus [36]. In DMD rats, the tongue showed hypertrophy of myofibers with less advanced dystrophic changes until 9 months old compared to masseter. This resistance against degeneration might be related to a higher transcription level of utrophin in tongue of wild-type and DMD rats compared to masseter of wild-type rats [37]. Increased expression of utrophin in the skeletal muscles of *mdx* mice results in a functional improvement in muscle function [38–40]. However, in *mdx* muscles spared from degeneration, as extraocular and intrinsic laryngeal muscles, the utrophin upregulation is unlikely to be a protective mechanism [41–43]. The extraocular muscles remain unaffected even in dystrophin-deficient utrophin double knockout mice [42]. In the intrinsic laryngeal muscles, the reduction of utrophin at 20 months in *mdx* mice did not instigate any degeneration in muscle fibers [43]. Further investigation of utrophin in partially spared tongue muscles throughout life span would be interesting, as utrophin and concomitant rescue of dystrophin-associated glycoproteins are irregularly correlated with other protected muscles.

The study of biomarkers in a muscle partially spared from the absence of dystrophin allows us to identify dystrophinopathy markers that change with age, regardless of muscle degeneration. Among the possible biomarkers analyzed, the metabolites alanine, methionine, 3-methylhistidine, taurine, and glycerol change with aging, but not between control and dystrophic whole tongue muscles. Thus, it is suggested that these biomarkers may be efficient for specific assessments, but care should be taken when using them to monitor the progression of the disease, since they change throughout life.

The analysis of the metabolic profile of tongue suggests that the muscle aging process has a high impact on its metabolism, regardless of the lineage, since it was possible to distinguish the different ages of animals of the same lineage (Fig. 3). High-resolution 1H NMR spectroscopy has been shown to differentiate skeletal muscle from adult and old mice. In addition, there is a general difference in composition between younger and older muscles in mice [44], and these results are confirmed in the scenario of dystrophinopathy.

It was not possible to distinguish the metabolic profile of the whole tongue between the wild-type and *mdx* strains. This result corroborates our histological analysis, which showed protection against fibrosis and myonecrosis in the mid-belly tongue of young and old dystrophic animals. The chosen metabolites (3-methylhistidine, acetate, alanine, creatine, glutamic acid, glycerol, glycine, isoleucine, methionine, phosphocreatine, succinic acid, and taurine) were altered between the wild-type and *mdx* animals in muscles that suffer degeneration, such as the diaphragm, quadriceps, and soleus [13, 20, 21]. Since these metabolites were not different between the strains in this study, it suggests that the protection previously observed in the tongue of 3-month-old adult animals [17] is also observed in young (1 month old) and old (21–25 months old) animals.

### Metabolite and protein analysis

Taurine is considered a biomarker for the aging of skeletal muscle in mice [44], corroborating the data from this project, since its concentration was higher in old dystrophic and non-dystrophic mice. Its decrease in young *mdx* mice, compared to wild type, has already been seen in other muscles, indicating the possibility of a deficient taurine synthesis by the *mdx* muscles [21]. In the tongue, this decrease occurs both in the *mdx* and in the wild type compared to older age, corroborating the protection of the tongue muscle in dystrophic pathology. Taurine has an osmoregulatory function that helps to balance intracellular Ca^2+^ levels, helping with cell integrity and membrane stability [13]. Kunert-Keil et al. (2014) [34] studied the differential expressions of genes involved in Ca^2+^ homeostasis in dystrophic masticatory muscles and found uneven expressions in the studied muscles. Despite being a priori protected muscle, in its study, the tongue of 100-day-old mice presented levels of expression of Ca^2+^ regulatory proteins typical of dystrophic muscles. Analyzing Ca^2+^ regulatory proteins at the age of 21 to 25 months old would provide a better view on the relationship of increased taurine, found in this study, with Ca^2+^ homeostasis in old mice. In addition, in muscle regeneration, there is an increase in taurine, regardless of the type of muscle or genetic etiology of the damage to the fiber, suggesting that metabolic changes are significant indicators of muscle status [45]. The level of taurine diminishes with advancing age, and reinstating this decline via taurine supplementation protects against age-related muscle mass loss and decreased functionality and also improves both health and lifespan across various species [46–48]. Its greater concentration in the old group tongue, in this project, suggests the activation of fiber metabolism to prevent muscle degeneration.

Glycerol is a component of triglycerides (fats and oils) and phospholipids [30]. In muscles affected by DMD, the glycerol concentration was increased in relation to the wild type, at 6 months of age, being one of the metabolites responsible for the differentiation between the dystrophic and non-dystrophic muscles [13]. The results of this work showed that glycerol is more concentrated in the tongue of old animals, both in wild-type and *mdx* mice, with no difference between strains, suggesting its relationship with the aging process in the tongue mice muscle.

Alanine is a nonessential amino acid resulting from the conversion of pyruvate or the breakdown of DNA and carnosine and anserine dipeptides [30]. It can be used as a fuel for gluconeogenesis directly from muscle tissue and therefore plays an important role in glucose homeostasis [49, 50]. With the progression of dystrophinopathy in affected muscles, there is an increase in energy expenditure [10, 51], for the incorporation of amino acids into new proteins [52]. Therefore, the lower concentration of alanine in the tongue muscle of old animals may indicate incorporation of alanine into proteins for processes of muscle maintenance and regeneration related to age, since the decrease is seen in old dystrophic and non-dystrophic mice.

Methionine is an essential amino acid, substrate for protein synthesis, necessary for normal mammalian growth and development [30]. Martins-Bach et al. (2012) [13] showed that methionine increased with aging in quadriceps muscle samples from control mice from 3 months to 6 months of age. Besides, it showed that methionine concentrations did not change for *mdx* samples. However, in the whole tongue muscles, with a greater interval between the ages studied, methionine changed for both strains, being more concentrated in younger animals. The growth of the animals can elucidate this difference between the concentrations of methionine throughout the aging of the mice. From the 26th day of life until the 150th–200th, approximately 7 months of age, the mice’s growth is observed, then remaining on a *plateau* until the end of life [53]. The high concentration of methionine at a young age in this study suggests growth in young animals. In older animals, which are not in the growth phase, the metabolite is decreased. Furthermore, this similarity of methionine concentrations between strains suggests that the tongue muscle in *mdx* is metabolically closer to wild type, since this metabolite participates in the regulation of the immune system, lipid metabolism, oxidative stress, and other metabolic regulation processes (for review, see [54]). New studies should be carried out to characterize the involvement of this metabolite in the metabolism of DMD.

3-Methylhistidine (3-MeH) is an amino acid present in actin and myosin. It has been determined that more than 90% of body 3-MeH is located in the skeletal muscle. When skeletal muscle is degraded, 3-MeH is released but is not reused for protein synthesis [55]; therefore, the urinary excretion of 3-MeH can be used to indicate muscle protein degradation [56]. Furthermore, there is a progressive loss of skeletal muscle mass and muscle strength with aging [57, 58]. The higher concentration of 3-MeH observed in the tongue of the young groups may reflect the decrease in muscle mass and the consequent reduction in 3-MeH concentrations seen in older muscles.

Muscle degeneration biomarkers, which in other studies proved to be efficient (acetic acid, phosphocreatine, isoleucine, succinic acid, creatine, TNF-α, and TGF-β protein) [13, 19, 23], did not show any difference with aging and in the analysis between strains. The TGF-β protein is actively involved in the proliferation of fibrous connective tissue in the skeletal muscles of patients and *mdx* mice [26, 27]. In previous studies, the levels of TGF-β have already been related to the presence of fibrous tissue in *mdx*’s respiratory muscles [23]. Spassov et al. [17] studied changes in the expression of collagen in the masticatory muscles in 100-day-old animals and concluded that there was no difference between the tongue of *mdx* and control groups. Our results corroborate theirs, due to the equality in the quantification of TGF-β in young animals indicating protection against tissue fibrosis of this muscle in the initial phase of the disease. In old animals, the levels of TGF-β were also equal between strains and suggest that this muscle protection against fibrosis continues until advanced stages of dystrophy in the mice, corroborating the histological results for fibrosis found in our study, for both ages.

TNF-α is a protein mediator of inflammatory response produced by activated macrophages, mast cells, endothelial cells, fibroblasts, myoblasts and myotubes, and injured myofibers [59, 60]. It stimulates the expression of adhesion molecules in endothelial cells, increasing leukocyte recruitment and adhesion. TNF-α is secreted at the site of inflammation and can enter the bloodstream [59]. The TNF-α is related to the progression of DMD, and its absence is related to muscle protection for the disease. Maranhão et al. [23] evidenced the progressive increase in the concentration of TNF-α in *mdx* diaphragm muscles, in comparison with the control and over age, with 1, 4, and 9 months. The intrinsic muscles of the larynx were identified as muscles spared from dystrophinopathy in the *mdx* mice, as they did not show signs of muscle damage, degeneration, or regeneration, during the course of the disease [24]. The concentrations of TNF-α in intrinsic muscles of the larynx were comparable to those of the control animal, during the progression of the disease, even in later stages, at 20 months of age [23]. These results corroborate with this work, since the concentrations of TNF-α in the tongue of the *mdx* animals did not differ from the wild-type animals, in young and old. Messina et al. (2011) [25] analyzed vastus lateralis muscle samples from DMD patients and showed an increase in the concentration of TNF-α in relation to the control group, besides pointing out the increase in its expression with age and disease progression, from 2 to 9 years of age. In this work, the concentration of TNF-α did not increase with aging in tongue in both strains, corroborating with the protection previously mentioned, including with disease progression and aging.

The metabolites glycine and glutamic acid were different only in dystrophic animals, being more concentrated in young *mdx* mice. Glycine is a nonessential amino acid involved in the production of DNA, phospholipids, and collagen, in addition to being involved in the release of energy [30]. Ham, Gardner, Kennedy, Trieu, Naim, Chee, Alves, Caldow, Lynch, and Koopman [61] demonstrated that glycine supplementation in *mdx* mice and dystrophin/utrophin double knockout mice can attenuate the progression of dystrophic pathology, as well as improve the effectiveness of prednisolone, the current gold standard treatment for DMD. Anderson and Skrabek [62] studied the heart of *mdx* mice treated with deflazacort, from a metabolomic perspective. They showed that the concentration of glycine in the *mdx* heart decreased with disease progression, but with a 2-week high-dose treatment with deflazacort, glycine levels increased above the levels of the *mdx* placebo group. Glycine supplementation has already been shown to protect against loss of myotubes in nutrient restricted/growth factor restriction models with C2C12 muscle cells in vitro. This in vitro protection has been shown to be dependent on mammalian target of rapamycin complex 1 (mTORC1) signaling [63] and also a specific glycine activation of mTORC1 involved in muscle regeneration in dystrophic mice [48, 64]. Thus, the protection observed in the 1-month-old tongue of the *mdx* muscle, close to the 20th day degeneration peak, may be related to the high levels of glycine presented in our study. In mdx and control mice, a gradual decline in muscle mass becomes apparent around 18 months, marking the onset of sarcopenia [65]. Mouisel and colleagues (2010) [66] have reported decreased levels of recovery of maximum force after myotoxic injury and of phosphorylated Akt and S6 (i.e., participants in the mTOR signaling pathway) in the tibialis anterior of 18–24-month-old *mdx* mice in comparison to 5-month-old *mdx* mice [64]. The decrease in regeneration of skeletal muscles from 18 months of age would explain the lower glycine levels in the tongue of old *mdx* found in this study, since a specific glycine activation of mTORC1 pathway is linked to muscle regeneration in dystrophic mice.

Glutamic acid, also known as glutamate (the anion), is a nonessential amino acid, one of the 20 proteinogenic amino acids and the most abundant rapid excitatory neurotransmitter in the nervous system [30]. Glutathione is the main cellular antioxidant and regulates free radical homeostasis. Glutamate plays an essential regulatory role in the synthesis of glutathione and is also related to its functions (for review, see [67, 68]). It is proven the relationship between decreased intracellular glutathione and stress states, such as chronic diseases [68], besides demonstrating benefits in the use of different antioxidant drugs for preclinical studies in *mdx* mice, such as improvement in dystrophinopathy with decreased necrosis [69]. In a metabolomic study of the biceps femoris muscle of the canine model for DMD (golden retriever muscular dystrophy), there was an increase in the concentration of glutamic acid when compared to the control [70]. Laferte, Rosenkrantz, and Berlinguet (1963) [71] observed an increase in weight loss and acceleration of the beginning of the terminal phase of the disease in mice that received exogenous glutamic acid. The increased glutamate concentration in the dystrophic muscles of the quadriceps and diaphragm suggests its connection with muscle regeneration and its requirement of protein replacement [13] and its relationship with the pathology of DMD. In this study, the amounts of glutamic acid in the tongue *mdx* muscles were equivalent to those of control muscles of the same age, indicating protection against myonecrosis on the tongue. As previously mentioned, skeletal muscle of older *mdx* mice (18-24-month-old) present sarcopenia with reduced regeneration, including reduced mTORC1 activation for muscle protein synthesis [66]. Thus, lower glutamate levels in old versus young *mdx* mice are expected. The observed decrease of glycine and glutamate levels suggests that mTOR-related pathways may not contribute to the protective mechanisms against the dystropathology seen in the old *mdx* tongue.

These results corroborate the protection observed in histology and the use of these as muscle biomarkers of degeneration and regeneration. Other studies on affected muscles can elucidate their specificities as biomarkers for the progression of DMD, without the interference of aging.

## Conclusions

The tongue of young (1 month) and old (21 to 25 months) *mdx* animals remains spared from the intense and progressive myonecrosis described in other muscles, as evidenced by mid-belly histological analysis. This protection was verified by the biomarker proteins for inflammation and fibrosis and by the concentrations of the metabolites 3-methylhistidine, acetic acid, glutamic acid, alanine, creatine, phosphocreatine, glycerol, glycine, isoleucine, methionine, succinic acid, and taurine already used for dystrophic muscle differentiation and in this work presented similar results between wild-type and *mdx* whole tongue muscles.

Among the possible biomarkers analyzed, the metabolites alanine, methionine, 3-methylhistidine, taurine, and glycerol may be efficient for specific assessments, but care should be taken when using them to monitor the progression of the disease, since they change throughout life.

Muscle degeneration biomarkers, which in other studies proved to be efficient (acetic acid, phosphocreatine, isoleucine, succinate, creatine, TNF-α, and TGF-β), did not show any difference with aging and in the spared muscles. Other studies on affected muscles can elucidate their specificities as biomarkers for the progression of DMD, without the interference of aging.

### Supplementary Information

**Additional file 1.** 

## Data Availability

Not applicable.

## Abbreviations

3-MeH: 3-Methylhistidine
ANOVA: Analysis of variance
CEUA: Brazilian College for Animal Experimentation
CN: Central nuclei
D2O: Deuterated water
DMD: Duchenne muscular dystrophy
FIB: Areas of fibrosis
GAPDH: Glyceraldehyde 3-phosphate dehydrogenase protein
*mdx*: X chromosome-linked muscular dystrophy
mTORC1: Mammalian target of rapamycin complex 1
NMR: Nuclear magnetic resonance
PN: Areas of muscle fibers with peripheral nuclei
iPCA: Interactive principal component analysis
TNF-α: Tumor necrosis factor alpha
TGF-β: Transforming growth factor beta
TSP: Trimethylsilyl tetradeuteropropionic acid
UNESP: São Paulo State University
