# Supplementary material for: Biomarkers for Duchenne muscular dystrophy progression: impact of age in the mdx tongue spared muscle
Source: Skelet Muscle. 2023 Sep 13;13:16. doi: 10.1186/s13395-023-00325-z (PMC10500803; doi:10.1186/s13395-023-00325-z)
Supplement: Supplementary file 1 — Additional file 1. [file 13395_2023_325_MOESM1_ESM.pdf]

! TITLE = Multi integration tool
! DATE = Tue 2020-01-14 14:51:32
! FILE = C:\Documents and Settings\gang\Desktop\Marcelo/result.txt
! USER = gang
! HOST = 1393-RAFTERYLPT
!
! dimension = 1D
! use reference spectra = no
! number of spectra to test = 31
! integration mode = sum all points in region
! multiplier = 1.000000
! use positive = no
! apply normalization = no

regions
number of regions: 13

left	right	
1.0283	1.0056	Isoleucine-3
1.5053	1.4639	Alanine-3
1.9262	1.9155	Acetic-acid-3
2.4134	2.4002	Succinic acid-4
3.0459	3.0253	Creatine-9
3.0533	3.0458	Phosphocreatine-9
3.4469	3.3964	Taurine-2
3.5648	3.5587	Glycine-2
2.6426	2.6372	Methionine-0.5
2.3754	2.3322	Glutamic acid-2
7.0516	7.0091	3-Methylhistidine-1
3.6507	3.639	Glycerol-1.16
0.1198	-0.1362	TSP-9

spectra	sum	Isoleucin	Ala	Aceti	Succin	Cre	Phosph	Tau	Gly	Methi	Glutar	3-Methy	Glyce	TSP-9	title
TON_wild_type_young_1	39942320512	114303514	2507083	9214406	9796768	9169740	2906778	1151386	6758607	4256293	1229507	1214873	3871857	11988930474	Water Suppression Spectrum
TON_wild_type_young_2	36652511926	74210118	2162386	8563267	8567297	8297524	2634567	1030473	4805593	3843731	9354442	9590232	4360657	11850733060	Water Suppression Spectrum
TON_wild_type_young_3	37751180202	98508794	2088510	8351373	1029364	8333688	1980746	1080524	4766576	4039935	9735992	9835972	3076514	12465979776	Water Suppression Spectrum
TON_wild_type_young_4	35807370194	101019902	2087727	8362270	5369421	7450712	2498200	1055301	5082697	4552359	1008844	8224568	3720953	11974924366	Water Suppression Spectrum
TON_wild_type_young_5	35236650968	145162302	2027838	7738968	7936099	6597103	2310666	1061072	4461333	4195589	9964063	1168259	3283732	12127548998	Water Suppression Spectrum
TON_wild_type_young_6	26167606840	63062726	1546058	5607546	4755415	5835900	2342253	8038824	5256583	3335212	8582871	8511856	2616953	8153806431	Water Suppression Spectrum
TON_wild_type_young_7	27054151386	66707574	1576752	1694563	4809298	4898481	2402183	7693610	4599517	2477650	8506781	6410180	2522940	10276192544	Water Suppression Spectrum
TON_wild_type_young_8	26102834917	79995620	1678857	1538337	7888676	4888847	2477656	7910828	4771813	2243202	6541476	8521028	2519970	8862870639	Water Suppression Spectrum

TON_wild_type_young_9	28150719198	70419256	1541683	7792181	4305893	6353899	2373857	9222815	4395760	2743993	8115216	6230636	2870105	8588150372	Water Suppression Spectrum
TON_wild_type_young_10	25789011246	64504644	1322742	2545364	3300804	5086165	2363589	8514189	3790952	2102333	7503419	7983358	2184016	8531736256	Water Suppression Spectrum
TON_mdx_young_1	38198709134	95537404	1792927	8857438	7246705	7755151	2110335	1247262	5755735	5363985	9744592	1000929	3675598	12189696674	Water Suppression Spectrum
TON_mdx_young_2	38393618090	87584852	1768999	8052637	5475274	8145170	2046826	1262571	6581327	2679732	9824628	6997988	3012191	12170080494	Water Suppression Spectrum
TON_mdx_young_3	39152134732	107008394	1858427	8339084	5826708	8181485	2262917	1272649	6707936	4032403	1098136	8616110	3253752	12415055224	Water Suppression Spectrum
TON_mdx_young_4	35658933032	95158380	1614824	9034644	5068647	7029487	2225244	1080073	5139736	4660265	1084396	8905368	2913899	12460462176	Water Suppression Spectrum
TON_mdx_young_5	42371683478	140185016	2145386	8954883	4894640	9103997	2445357	1447729	6502599	4230679	1450226	1014919	3125423	12318501068	Water Suppression Spectrum
TON_mdx_young_6	38072920996	114285592	1919380	8168084	3434148	8380920	2273052	1173236	8094119	4595142	1230318	8256178	3553360	12014866264	Water Suppression Spectrum
TON_mdx_young_7	25482766246	56929812	1617530	2353030	6723588	5230115	2145083	7825407	3934314	3993388	6974212	6526729	2338393	8200720329	Water Suppression Spectrum
TON_mdx_young_8	29135942365	82016952	1944910	3498233	2405620	5682709	3104187	1040522	4250090	3119920	7708454	6811627	3030337	8522075278	Water Suppression Spectrum
TON_mdx_young_9	26157013937	56158928	1379121	6633380	7088744	5347076	1677505	8136256	4097710	3172855	7338393	7269249	2454588	8801952107	Water Suppression Spectrum
TON_mdx_young_10	24710743655	68509302	1274031	5722216	4645689	5207559	1762267	7530249	4030020	3233712	7237681	5904867	2021099	8512110669	Water Suppression Spectrum
TON_mdx_young_11	26280033055	64958437	1485641	9719033	4253634	5893102	1951321	8213223	4384760	3473779	6948923	6127766	2096312	8466406526	Water Suppression Spectrum
TON_wild_type_old_1	33341917114	81031238	1279149	2132087	2781198	7179155	2171974	1332750	3830750	2419933	9238650	5435280	6738421	8707217442	Water Suppression Spectrum
TON_wild_type_old_2	29639328196	65142750	1215814	8001347	5563798	6412365	2138698	1093272	2850115	1773924	7917719	3905994	5362073	8493228595	Water Suppression Spectrum
TON_wild_type_old_3	23370708243	55149819	1026924	1809221	6189028	3631932	1692889	8046790	2674953	9674340	3276793	4519907	4657758	8524972970	Water Suppression Spectrum
TON_wild_type_old_4	32028306197	62780570	1297220	9719826	5063451	7513689	2740104	1170586	3664883	1818298	7567703	4619600	4395925	8943962185	Water Suppression Spectrum
TON_wild_type_old_5	27650758121	62794525	1153946	8509883	3061701	5117744	2946117	1008923	3403364	1668847	6432724	4913907	4851983	9006525426	Water Suppression Spectrum
TON_mdx_old_1	24217914733	52676027	9952156	1658906	6604638	4420972	1658995	8677877	2237035	1290699	2996227	4453952	5388438	7959302058	Water Suppression Spectrum
TON_mdx_old_2	27309083640	69964494	1131701	1873197	5653793	5114593	1750223	1020678	3009742	1617996	5963263	4935483	4637882	8431698176	Water Suppression Spectrum
TON_mdx_old_3	25770053681	60922875	1003893	6612963	5184043	4406912	2223179	9695425	2484855	1528557	5211443	4359623	4714866	8496049222	Water Suppression Spectrum
TON_mdx_old_4	27714603618	113776065	8466112	2445381	2353748	5504992	1794134	1085431	2899507	1632122	4788860	5153856	4500689	8448817894	Water Suppression Spectrum
TON_mdx_old_5	27822353301	111525225	9816676	2308614	1905983	5885570	1917629	1032261	3922837	1585685	5000738	4395474	4389374	8516646637	Water Suppression Spectrum

Results in Micromolar

spectra	Isoleucin	Ala	Aceti	Succin	Cre	Phosph	Tau	Gly	Methi	Glutar	3-Methy	Glyce	TSP-9
TON_wild_type_young_1	14.30113148	313.6748	115.2864	91.92950	1147.275	36.36827	2160.842	126.8408	31.95167	230.7455	45.59982	125.2833	500
TON_wild_type_young_2	9.39310475	273.7029	108.3890	81.33007	1050.254	33.34689	1956.474	91.23980	29.19109	177.6050	36.41635	142.7451	500
TON_wild_type_young_3	11.85331548	251.3052	100.4899	92.89563	1002.771	23.83382	1950.252	86.03251	29.16691	175.7261	35.50613	95.73861	500
TON_wild_type_young_4	12.65392986	261.5123	104.7472	50.44373	933.2892	31.29290	1982.834	95.50013	34.21418	189.5545	30.90671	120.5413	500
TON_wild_type_young_5	17.95444842	250.8138	95.71969	73.61843	815.9650	28.57955	1968.587	82.77023	31.13597	184.8612	43.34897	105.0386	500
TON_wild_type_young_6	11.60121838	284.4178	10.31581	65.61159	1073.590	43.08882	2218.271	145.0526	36.81336	236.8398	46.97603	124.5059	500
TON_wild_type_young_7	9.737201845	230.1561	24.73528	52.65044	715.0237	35.06430	1684.536	100.7076	21.69952	186.2582	28.07052	95.24217	500
TON_wild_type_young_8	13.53888992	284.1388	26.03564	100.1341	827.4149	41.93320	2008.306	121.1411	22.77910	166.0672	43.26434	110.3000	500
TON_wild_type_young_9	12.29937524	269.2692	13.60976	56.40480	1109.767	41.46162	2416.275	115.1640	28.75583	212.6096	32.64714	129.6440	500
TON_wild_type_young_10	11.34082947	232.5568	44.75111	43.52461	894.2199	41.55525	2245.372	99.97547	22.17720	197.8811	42.10762	99.30543	500
TON_mdx_young_1	11.75633076	220.6282	108.9949	66.88061	954.3081	25.96867	2302.223	106.2405	39.60383	179.8677	36.95073	116.9740	500
TON_mdx_young_2	10.79510346	218.0347	99.25124	50.61333	1003.917	25.22777	2334.237	121.6753	19.81711	181.6373	25.87571	96.01601	500
TON_mdx_young_3	12.92886645	224.5371	100.7536	52.79917	988.4956	27.34080	2306.443	121.5689	29.23195	199.0170	31.23022	101.6694	500
TON_mdx_young_4	11.45523882	194.3938	108.7597	45.76257	846.2150	26.78766	1950.300	92.80880	33.66037	195.8107	32.16105	90.71830	500
TON_mdx_young_5	17.0700577	261.2395	109.0418	44.70081	1108.576	29.77663	2644.308	118.7713	30.90969	264.8869	37.07542	98.42501	500
TON_mdx_young_6	14.26802298	239.6256	101.9747	32.15530	1046.318	28.37800	2197.095	151.5769	34.42092	230.3992	30.92236	114.7294	500

TON_mdx_young_7	10.41307526	295.8636	43.03945	92.23625	956.6443	39.23589	2147.026	107.9442	43.82602	191.3487	35.81427	110.6165	500
TON_mdx_young_8	14.43608792	342.3305	61.57361	31.75661	1000.233	54.63787	2747.188	112.2109	32.94887	203.5187	35.96814	137.9431	500
TON_mdx_young_9	9.570421536	235.0253	11.30439	90.60305	911.2313	28.58749	2079.831	104.7477	32.44246	187.5877	37.16405	108.1818	500
TON_mdx_young_10	12.07267586	224.5091	10.08366	61.39958	917.6735	31.05458	1990.465	106.5252	34.19058	191.3131	31.21658	92.10959	500
TON_mdx_young_11	11.50873812	263.2122	17.21928	56.52148	1044.085	34.57171	2182.715	116.5277	36.92713	184.6719	32.56983	96.05310	500
TON_wild_type_old_1	13.95932257	220.3602	36.72965	35.93396	1236.759	37.41679	3443.910	98.98901	25.01304	238.7325	28.09021	300.2156	500
TON_wild_type_old_2	11.50494466	214.7264	14.13128	73.69721	1132.496	37.77183	2896.263	75.50437	18.79770	209.7537	20.69527	244.9144	500
TON_wild_type_old_3	9.703811237	180.6911	31.83390	81.67365	639.0516	29.78700	2123.793	70.60016	10.21341	86.48456	23.85882	211.9524	500
TON_wild_type_old_4	10.52898626	217.5580	16.30120	63.68970	1260.127	45.95453	2944.802	92.19614	18.29690	190.3779	23.24272	190.6667	500
TON_wild_type_old_5	10.4581715	192.1851	14.17286	38.24353	852.3395	49.06637	2520.480	85.02246	16.67638	160.7015	24.55173	208.9857	500
TON_mdx_old_1	9.927257431	187.5570	31.26353	93.35263	833.1709	31.26522	2453.132	63.23832	14.59461	84.69978	25.18158	262.6288	500
TON_mdx_old_2	12.4466909	201.3297	33.32419	75.43578	909.8867	31.13650	2723.681	80.31503	17.27050	159.1297	26.34069	213.3826	500
TON_mdx_old_3	10.75609499	177.2400	11.67536	68.64424	778.0521	39.25082	2567.629	65.80618	16.19224	138.0141	23.09109	215.2816	500
TON_mdx_old_4	20.19976044	150.3070	43.41521	31.34127	977.3544	31.85300	2890.606	77.21661	17.38598	127.5318	27.45041	206.6510	500
TON_mdx_old_5	19.64245373	172.8968	40.66062	25.17694	1036.599	33.77436	2727.115	103.6368	16.75678	132.1137	23.22467	199.9348	500
